# Supplementary material for: Epidemiological characterization of ischemic heart disease at different altitudes: A nationwide population-based analysis from 2011 to 2021 in Ecuador
Source: PLoS One. 2023 Dec 29;18(12):e0295586. doi: 10.1371/journal.pone.0295586 (PMC10756509; doi:10.1371/journal.pone.0295586)
Supplement: S1 Table — (PDF) [file pone.0295586.s002.pdf]

Supplementary Table 1 Displays the incidence and mortality rates of ischemic heart disease in Ecuador from 2011 to 2021 according to different age groups and gender. The table shows that both incidence and mortality rates increased with age and were generally higher in men than in women. The highest incidence rate was observed in the age group >80, with an incidence rate of 210.29/100,000 in women and 348.32/100,000 in men. The highest mortality rate was observed in the same age group, with a mortality rate of 1232.04/100,000 in women and 1398.48/100,000 in men.

	**Women**			**Men**		
**Age (years)**	**Cases n**	**Incidence Rate**	**Mortality Rate**	**Cases n**	**Incidence Rate**	**Mortality Rate**
**< 1**	NA	NA	NA	2	0.58 (0.58 - 0.58)	0.93 (0.76 - 1.11)
**1 - 4**	4	0.6 (NA)	0.15 (NA)	4	0.58 (NA)	0.15 (NA)
**5 - 9**	5	0.31 (0.24 - 0.38)	0.12 (0.12 - 0.12)	2	0.24 (NA - NA)	0.12 (NA - NA)
**10 - 14**	4	0.17 (0.13 - 0.21)	0.19 (0.11 - 0.26)	7	0.44 (0.22 - 0.65)	0.24 (0.1 - 0.38)
**15 - 19**	43	0.51 (0.47 to 0.55)	0.83 (0.77 to 0.89)	70	0.8 (0.77 to 0.84)	1.72 (1.62 to 1.82)
**20 - 24**	47	0.6 (0.55 to 0.64)	0.91 (0.83 to 0.98)	142	1.79 (1.72 to 1.86)	3.53 (3.32 to 3.73)
**25 - 29**	60	0.82 (0.75 to 0.88)	1.47 (1.36 to 1.58)	222	3.08 (2.95 to 3.21)	4.98 (4.71 to 5.25)
**30 - 34**	98	1.44 (1.35 to 1.53)	2.4 (2.26 to 2.55)	365	5.61 (5.5 to 5.71)	7.88 (7.42 to 8.34)
**35 - 39**	189	3.03 (2.91 to 3.15)	3.86 (3.5 to 4.22)	617	10.61 (10.33 to 10.88)	11.15 (10.41 to 11.89)
**40 - 44**	331	5.93 (5.74 to 6.12)	6.67 (6.17 to 7.16)	1,050	20.42 (19.82 to 21.01)	19.11 (17.67 to 20.55)
**45 - 49**	573	11.61 (11.31 to 11.91)	11.57 (10.58 to 12.56)	1,898	41.68 (40.43 to 42.93)	27.32 (25.42 to 29.22)
**50 - 54**	952	22.35 (21.98 to 22.72)	18.88 (17.49 to 20.26)	3,197	81.04 (79.55 to 82.52)	46.66 (43.54 to 49.78)
**55 - 59**	1,379	38.43 (37.3 to 39.55)	34.35 (31.16 to 37.53)	4,651	139.63 (136.86 to 142.4)	79.67 (74.08 to 85.26)
**60 - 64**	1,781	61.44 (59.81 to 63.06)	59.33 (53.7 to 64.97)	5,556	207.4 (201.49 to 213.31)	132.38 (122.22 to 142.53)
**65 - 69**	1,959	85.75 (83.05 to 88.45)	93.8 (84.37 to 103.22)	5,550	267.26 (259.25 to 275.28)	192.26 (176.27 to 208.25)
**70 - 74**	2,003	115.9 (112.84 to 118.95)	155.97 (142.44 to 169.51)	4,457	290.96 (279.94 to 301.97)	298.79 (271.47 to 326.12)
**75 - 79**	1,806	147.47 (143.35 to 151.58)	273.03 (248.73 to 297.33)	3,566	337.63 (327.17 to 348.09)	476.12 (434.14 to 518.1)

Age (years)	Women			Men		
	**Cases n**	**Incidence Rate**	**Mortality Rate**	**Cases n**	**Incidence Rate**	**Mortality Rate**
**> 80**	2,982	210.29 (204.54 to 216.04)	1232.04 (1120.44 to 1343.65)	4,193	348.32 (338.12 to 358.53)	1398.48 (1260.67 to 1536.29)
**Total**	14,216	20.54 (18.27 to 22.81)	44.53 (34.45 to 54.61)	35,555	54.53 (49.1 to 59.96)	68.31 (56.66 to 79.96)

6
