# Supplementary material for: Epidemiological characterization of ischemic heart disease at different altitudes: A nationwide population-based analysis from 2011 to 2021 in Ecuador
Source: PLoS One. 2023 Dec 29;18(12):e0295586. doi: 10.1371/journal.pone.0295586 (PMC10756509; doi:10.1371/journal.pone.0295586)
Supplement: Attachment — Submitted filename: Review Report .docx [file pone.0295586.s004.docx]

**Review Report**

Manuscript title:

"Epidemiological Characterization of Ischemic Heart Disease at Different Altitudes: A Nationwide Population-Based Analysis from 2011 to 2021 in Ecuador"

General Overview

An epidemiological analysis of ischemic heart disease (IHD) at various altitudes in Ecuador is presented in this manuscript. Due to the country's geographic diversity, this approach is both innovative and relevant.

Responses to previous comments

- In terms of confounding variables, the authors have adequately addressed concerns, although they acknowledge the limitation of not including some, such as lifestyle habits and genetic factors. As a result, the study becomes more transparent.
- The impact of tourism and mobility on incidence rates has been effectively addressed by clarifying that the place of residence was used for calculation rather than the place of medical care.
- The authors have included a more detailed methodology section, including exclusion criteria, which makes the study clearer.

Importan annotations

1. Despite the authors' efforts to correct several inconsistencies in table and figure labels, the consistency and accuracy of data presentation throughout the manuscript is crucial.
2. The authors should ensure that the interpretation of the results and conclusions are consistent with the presented data. Avoid making claims that are not supported by the data.
3. While a limitations section has been added, it would be beneficial to delve deeper into how these limitations might affect interpretations and applications of the results. A more detailed discussion of this aspect would enhance the validity of the study.
4. The authors should suggest specific directions for future research, especially in areas where the current study has limitations. There might be longitudinal studies or more detailed analyses of specific risk factors at different altitudes.

Conclusion

This manuscript provides significant insights into the relationship between IHD and altitude in Ecuador. In response to previous comments, the authors have improved the clarity and quality of the study. It is, however, crucial to maintain a critical and reflective attitude when interpreting data and drawing conclusions. It would be beneficial to include suggestions for future research based on the limitations of the current study.
